# Design and Evaluation of a Poly(Lactide-*co*-Glycolide)-Based In Situ Film-Forming System for Topical Delivery of Trolamine Salicylate

**DOI:** 10.3390/pharmaceutics11080409

**Published:** 2019-08-12

**Authors:** Yujin Kim, Moritz Beck-Broichsitter, Ajay K. Banga

**Affiliations:** 1 Centre for Drug Delivery Research, Department of Pharmaceutical Sciences, College of Pharmacy, Mercer University, Atlanta, GA 30341, USA; 2 MilliporeSigma a Business of Merck KGaA, Frankfurter Strasse 250, 64293 Darmstadt, Germany

**Keywords:** NSAIDs, PLGA, polymeric film, topical drug delivery, trolamine salicylate

## Abstract

Trolamine salicylate (TS) is a topical anti-inflammatory analgesic used to treat small joint pain. The topical route is preferred over the oral one owing to gastrointestinal side effects. In this study, a poly(lactide-*co*-glycolide) (PLGA)-based in situ bio-adhesive film-forming system for the transdermal delivery of TS was designed and evaluated. Therefore, varying amounts (0%, 5%, 10%, 20%, and 25% (*w*/*w*)) of PLGA (EXPANSORB^®^ DLG 50-2A, 50-5A, 50-8A, and 75-5A), ethyl 2-cyanoacrylate, poly (ethylene glycol) 400, and 1% of TS were dissolved together in acetone to form the bio-adhesive polymeric solution. In vitro drug permeation studies were performed on a vertical Franz diffusion cell and dermatomed porcine ear skin to evaluate the distinct formulations. The bio-adhesive polymeric solutions were prepared successfully and formed a thin film upon application in situ. A significantly higher amount of TS was delivered from a formulation containing 20% PLGA (45 ± 4 µg/cm^2^) and compared to PLGA-free counterpart (0.6 ± 0.2 µg/cm^2^). Furthermore, the addition of PLGA to the polymer film facilitated an early onset of TS delivery across dermatomed porcine skin. The optimized formulation also enhanced the delivery of TS into and across the skin.

## 1. Introduction

Topical drug delivery has many advantages over systemic drug administration, such as minimizing side effects, bypassing first-pass metabolism, and ensuring better patient compliance [1]. However, dermal drug delivery is usually limited to certain drug molecules, owing to the well-known barrier function of the skin. The outermost layer of the skin, the stratum corneum, generally only allows permeation of molecules with a molecular weight <500 Da, a log*P* between 1 and 3, and a melting point of <250 °C [2].

Among the available topical formulations, non-steroidal anti-inflammatory drugs (NSAIDs) constitute approximately 18 world-wide marketed molecules (some examples include diclofenac, methyl salicylate, salicylic acid, and ketoprofen) [3]. As an example, topical salicylates are known to be absorbed by the dermal tissue and have been reported to be effective in relieving local pain [4]. Trolamine salicylate (TS), a derivative of salicylic acid offers many benefits over other topical analgesics, including a lack of distinct odor, low systemic absorption upon dermal or topical administration, and low skin irritation [5]. Although topical salicylates are widely used, compliance is often an issue, owing to the frequent dosing regimen (three to four times per day).

The development of topical formulations has made an important contribution to medical practice. To deliver active pharmaceutical ingredients into or through the skin, various types of formulations are known, such as gels and emulsions. The type of vehicle chosen depends on the properties of the drug and the intended target area. In addition, the hydrophilicity and lipophilicity of the drug must be compatible with the vehicle. “Conventional” vehicles are inelegant and have the drawback of poor control of both the amount of drug applied and the area of skin exposed [6]. As a result, the use of topical formulations to deliver molecules to systemic circulation is less than ideal, thus resulting in substantial variability in the extent and duration of drug effects [7]. To overcome the shortcomings of skin drug delivery, the former Alza Corporation pioneered a transdermal patch in which system design and explicit control of the surface area led to improved passive drug delivery to the systemic circulation at a predetermined rate. Transdermal patches are recommended to be applied to a flat surface area to ensure adhesion to skin over a prolonged period of time. However, in the case of arthritis and other joint pain, the use of a patch is not suitable because of the uneven surface at the application sites. Pain associated with arthritis requires drug application at the pain region, which is usually difficult to cover with conventional patches [8]. In addition, clinical trials have shown that the topical application of NSAIDs delivers drugs at a higher local concentration to the tissue, resulting in better pain management [9].

Bioadhesive in situ film-forming systems provide many advantages such as higher dosing flexibility, extended release properties, higher patient compliance, improved cosmetic appearance, and less chance for loss of formulation by rubbing [10].

Poly(lactide-*co*-glycolide) (PLGA) is one of the most promising polymers used for the fabrication of drug delivery devices and tissue engineering applications [11]. PLGA is biocompatible and biodegradable, and its properties such as the erosion profile and mechanical strength can be controlled as needed. Furthermore, PLGA can be engineered to control the drug release behavior by changing the polymer molecular weight and the molar ratio of lactide to glycolide [12,13,14]. However, a detailed characterization of such systems is required to prevent potential dose dumping, and an inconsistent drug release profile [15].

In this study, a PLGA-based in situ bioadhesive film-forming system for the transdermal delivery of TS was designed and evaluated for drug permeation across dermatomed porcine ear skin. Different types of plasticizer and solvents were screened to optimize the film forming behavior. The effects of different ingredients were studied on the permeation profile of TS. The optimized formulations were developed by finding a proper ratio between polymer, adhesive, and plasticizer and showed enhancement of TS delivery into and across the skin.

## 2. Materials and Methods

### 2.1. Materials

All polymers (Table 1) were kindly donated by Merck KGaA (Darmstadt, Germany). Trolamine salicylate and ethyl-2-cyanoacrylate were purchased from Sigma (St. Louis, MO, USA). Glycerol and Poly(ethylene glycol) 400 was purchased from VWR (Richmond, BC, Canada) and Acros organics (Morris Plains, NJ, USA), respectively. Propylene glycol was obtained from EKI Industries (Joliet, IL, USA). Porcine ear skin was procured from a local slaughterhouse. Phosphate-buffered saline (PBS; 10 mM, pH 7.4), acetonitrile, ethyl acetate, and acetone were purchased from Fisher Scientific (Waltham, MA, USA).

### 2.2. Development of Film-Forming Polymeric Solutions

#### 2.2.1. Screening of Components

To choose a compatible solvent for PLGA, 1 g of PLGA and 0.25 g of cyanoacrylate (CA) were dissolved together in 1 g of four different solvents (ethanol, ethyl acetate, propylene glycols, and acetone) and left overnight mixing. To select a proper plasticizer, we added a few drops (0.25 g) of one of the three different plasticizers namely glycerol, poly(ethylene glycol) 400 (PEG 400), and propylene glycol to the polymeric mixture to increase the flexibility of the film. Each formulation (6.4 µL) was then applied on the dermatomed porcine ear skin, and the film forming behavior was investigated.

#### 2.2.2. Optimization of Formulations

Optimized film-forming solutions were prepared by the addition of the PLGA and CA to the solvent. After obtaining a clear solution, the plasticizer and the drug were added. The solutions were left overnight mixing in glass vials to allow the drug to dissolve completely at room temperature. A detailed composition of each formulation can be found in Table 2.

#### 2.2.3. Effect of Plasticizer PEG 400, Types of PLGA, and Various Concentration of PLGA for Topical Delivery of TS

PLGA, 50-2A, was used to test the effects of plasticizer on the permeation profiles of TS. The ratio of PLGA to CA was kept at four to one. Then, the proportion of the plasticizer was increased from 0 to 3. The highest amount PEG 400 was added to F7, at 11.25 g (11.25% (*w*/*w*)), then reduced to 8.75 g (F6; 8.75% (*w*/*w*)), 5 g (F’1; 5% (*w*/*w*)), and finally to no PEG 400 in F5 (Table 2).

For the comparison of different types of PLGA, the ratio of each component was kept at 4:1:1 (PLGA:cyanoacrylate:PEG 400). To investigate the effects of different amounts of PLGA in TS permeation profiles, we incorporated different amounts of PLGA into formulations. PLGA 50-2A was used in all formulations, and the ratio of PEG 400 and cyanoacrylate was kept at 1:1. To increase the amount of PLGA, the amount of cyanoacrylate was decreased from 25 to 0 in increments of 5.

### 2.3. Evaluation of the Formulations

#### 2.3.1. Crystallization Study and Solvent Evaporation Study

To observe the crystallization of the drug in the films, we placed 6.4 µL of formulations on a microscopic slide and evaluated the samples under a microscope after the evaporation of acetone. To study the extent and time of solvent evaporation, 6.4 µL of F1 formulation was placed on a microscopic slide using a positive displacement pipette. The slides were left in a convection oven (32 °C) for a pre-determined time and weighed at 2 min, 1 h and 24 h to calculate the percentage evaporation of the solvent over time.

#### 2.3.2. In Vitro TS Release Study

The release profiles of TS from the formulations F1, F2, F3, and F4 (different PLGA types) were determined to study the impact of the polymer properties on the drug release kinetics. Polymer films containing 64 µg of TS (equal to a surface area of 0.64 cm^2^) was added to the bottom of a glass vial. Then, 5 mL of PBS were added and left under constant shaking at 150 rpm and 30 °C. Samples (300 µL) were withdrawn from the vials at predetermined time points, and the same amount of fresh receptor solution was replaced. The samples were analyzed by high-performance liquid chromatography (HPLC) as outlined below.

#### 2.3.3. In Vitro Permeation Study

##### Skin Preparation

The outer region of full-thickness ear skin was removed carefully with a scalpel. The skin was then washed with PBS, dried, wrapped with Parafilm, and stored at −80 °C until use. The porcine ear skin was dermatomed with a Dermatome 75 mm (Nouvag AG, Goldach, Switzerland) prior to use. The average thickness of the dermatomed skin pieces was 0.50 ± 0.02 mm.

##### Evaluation of Skin Integrity

Before conducting a permeation study, we evaluated the integrity of the skin membrane by measuring the skin resistance value. The skin was mounted on a vertical Franz diffusion cell with the stratum corneum side facing up, and then 300 µL of PBS was added to the donor compartment. A silver electrode and silver chloride electrode were placed in the receptor and the donor chambers, respectively, without touching the skin membrane. Two electrodes were connected to a digital multimeter (34410A 6 ½ digit multimeter; Agilent Technologies, Santa Clara, CA, USA) and waveform generator (Agilent 33220A, 20 MHz function/arbitrary waveform generator) [16]. The resistance of the employed skin (R_s_) was calculated according to the following formula:

R_s_ = V_s_R_L_/(V_0_ − V_s_) (1)

where R_L_ and V_0_ were 100 kΩ and 100 mV, respectively. Skin pieces with a resistance lower than 10 KΩ were discarded.

Next, permeation studies were performed on a jacketed Franz diffusion cell with a diffusion area of 0.64 cm^2^ (PermeGear, Bethlehem, PA, USA) on dermatomed porcine ear skin. PBS was used as the receptor solution (5 mL). The skin was clamped between the donor and receptor chambers of a vertical diffusion cell with the stratum corneum side in contact with the donor solution. The distinct formulations (64 µg of TS per 0.64 cm^2^) was added in the donor compartment. The temperature of the receptor medium was maintained at 37 °C, and the skin surface temperature was about 32 °C. The amount of drug diffused over the dermatomed porcine ear skin was determined by removal of aliquots of 300 µL at pre-determined time points over 72 h from the receptor compartment. The volume was immediately replaced with the same amount of fresh buffer. The samples were analyzed by HPLC as outlined below. A skin extraction study was carried out to determine the drug amount that had penetrated into the skin. After 72 h, the skin samples were removed from Franz diffusion cell and the residual polymer film was removed with D-squame tape. The skin surface was wiped off with two cotton buds dipped in receptor solution. Then, the skin was dried with two cotton buds. The utilized tape and the four cotton buds were pooled and extracted with 30 mL of receptor solution. After the cleaning procedure, the epidermis and dermis were separated. The tissue was minced manually and added to 1 mL of receptor solution. Samples were shaken for 4 h followed by filtration using a 0.45 µm membrane filter and analyzed by HPLC.

### 2.4. Quantitative Analysis

TS was quantified by HPLC analysis. A Waters Alliance HPLC system (2795 Separating Module; Waters Co., Milford, MA, USA) equipped with a Photodiode detector (Waters 2475) and a Kinetex EVO C18 column (5 µm, 100 Å, 150 × 4.6 mm; Phenomenex, Torrance, CA, USA) was used. The mobile phase consisted of a 70/30 (*v*/*v*) mixture of acetonitrile and 0.1% of trifluoroacetic acid in distilled water. The injection volume and the flow rate were set to 20 µL and 1.0 mL/min, respectively, and the detection wavelength of TS was 304 nm. The reversed-phase HPLC method provided a linear range of 0.1–100 µg/mL (*R*^2^ = 0.999).

### 2.5. Statistical Analyses

All results are reported as the mean with the standard error of the mean (SE) from at least three replicates. Statistical calculations were performed with GraphPad Prism Version 8.0 (GraphPad Software, Inc., San Diego, CA, USA). One-Way ANOVA followed by Tukey HSD post hoc test was applied to compare the results of different groups. Statistically significant differences were denoted by *p* < 0.05.

## 3. Results

### 3.1. In Situ Film-Forming Behavior

PLGA and CA did not dissolve completely in propylene glycol and ethanol. Complete dissolution was achieved in ethyl acetate and acetone. However, ethyl acetate did not evaporate upon application and the obtained polymer films were difficult to handle. Therefore, acetone was chosen as the solvent for the system. A successful film formation behavior on the dermatomed porcine ear skin was observed with the plasticizer PEG 400. Upon administration, the solvent evaporated instantaneously and left a thin and transparent film layer behind.

### 3.2. Crystallization Study and Solvent Evaporation Study

**Figure 1:**
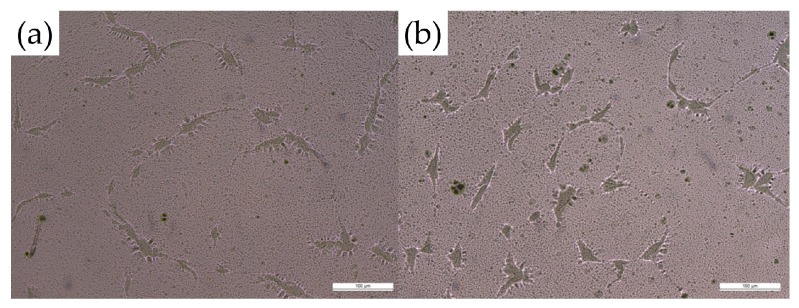
Microscopic images of the 20% PLGA formulation (F1) on glass sides (**a**) without and (**b**) with added drug (10× magnification) did not show drug crystallization after complete evaporation of the solvent.

Formulation (F1–F11) was observed under the light microscope after complete evaporation of the solvent. No drug crystals were observed in the polymer films during the slide crystallization study, indicating the solubility of TS in the film matrix after evaporation of the organic solvent (Figure 1). After 2 min in the convection oven, the result demonstrated 75.3 ± 4.1% (*n* = 3) solvent evaporation, and it did not further evaporate for 24 h.

### 3.3. In Vitro Release Study for Different Types of PLGA

**Figure 2:**
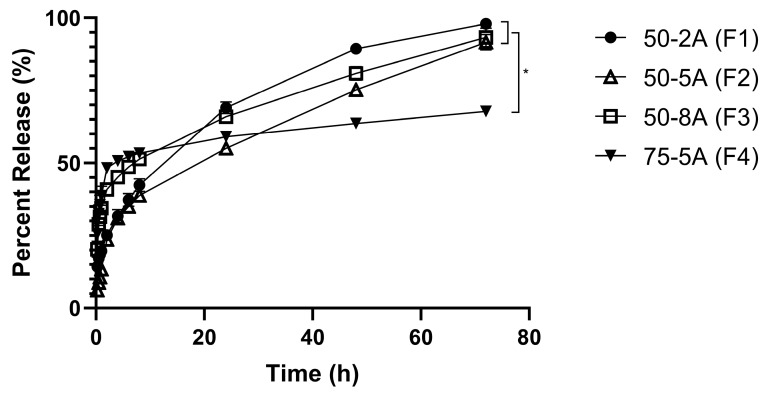
In vitro drug release profile from different types of PLGA (*n* = 6).

In the first hour, F3 and F4 released 34% and 38%, respectively. In contrast, a smaller amount was released after 1 h with F1 and F2 (20% and 13%, respectively). After 72 h, the drug release from F1, F2, and F3 was completed (>90%), whereas only 68% of TS was released from F4 (Figure 2).

### 3.4. Effects of Each Ingredient and Permeation Study

#### 3.4.1. Effects of Plasticizer

**Figure 3:**
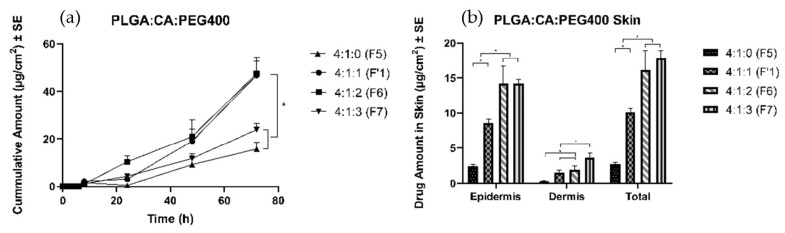
Permeation profiles of TS through porcine ear skin to study the effects of plasticizer. The group is representative of the ratio between PLGA:CA:PEG 400. (**a**) Cumulative amount (**b**) Average amount in the epidermis, dermis, and total skin.

Four formulations with different amounts of PEG 400 were prepared, and a thin film was formed upon application on the skin. The amount of TS delivered in the receptor solution after 72 h was found to be 16 ± 3 µg/cm^2^ and 24 ± 3 µg/cm^2^ in F5 and F7, respectively. A significantly higher amount of TS was delivered to the receptor with F1′ (47 ± 8 µg/cm^2^) and F6 (47 ± 5 µg/cm^2^). Moreover, formulation without any plasticizer (F5) delivered only 2.7 ± 0.3 μg/cm^2^, the least amount of drug into the skin (Figure 3). The highest amount of PEG 400 facilitated the largest amount of TS delivery into the skin (individual values in Table 3). Because there was no significant difference between the F1′ and F6 groups in the amount of drug in the receptor, the F1′ ratio was chosen to carry out permeation studies to investigate the effects of different types of PLGA.

#### 3.4.2. Effects of Different Types of PLGA

**Figure 4:**
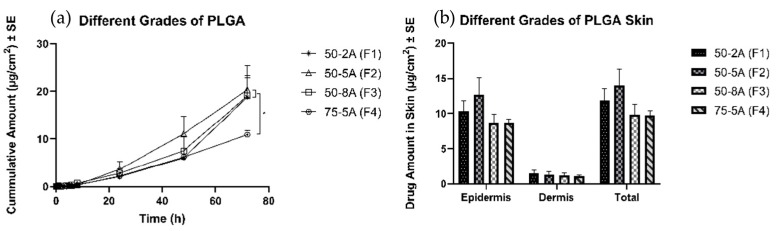
Permeation profiles of TS through porcine ear skin for the different types of PLGA. The group is representative of different types of PLGA: (**a**) Cumulative amount (**b**) Average amount in the epidermis, dermis, and total skin.

All formulations were able to form a polymeric solution, and then a thin layer of film was formed upon application on the skin. After 72 h, all three formulations with the one to one ratio (G/L) delivered 17 ± 3, 17 ± 3, and 19 ± 3 µg/cm^2^, respectively, and no significant difference was observed in the cumulative amount of TS in the receptor. In contrast, F4 delivered 11 ± 1 µg/cm^2^, a significantly lower amount than was observed for the other three groups. There was no significant difference in the drug amount delivered into the skin (Figure 4).

#### 3.4.3. Effects of Concentration of PLGA

**Figure 5:**
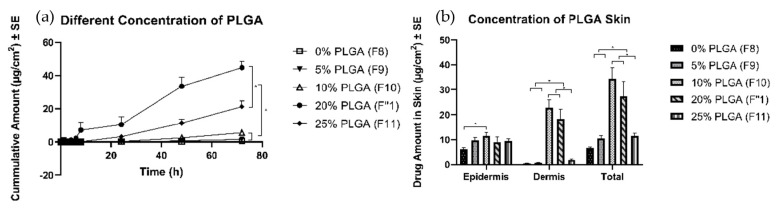
Permeation profiles of TS through porcine ear skin for different concentrations of PLGA. The group is representative of different concentrations of PLGA: (**a**) Cumulative amount (**b**) Average amount in the epidermis, dermis, and total skin.

All formulations were able to form a polymeric solution and formed a thin layer of film after complete evaporation of the solvent. The highest amount of TS (45 ± 4 µg/cm^2^) was delivered with 20% PLGA (F1″), followed by the formulation with 25% of PLGA (F11) (21 ± 4 µg/cm^2^). The formulations with 0%, 5%, and 10% PLGA delivered a significantly lower amount of TS, at 0.6 ± 0.2, 1.6 ± 0.3, and 5.6 ± 0.7 µg/cm^2^, respectively (Figure 5). The formulation with 10% (F10) and 20% (F1″) PLGA delivered significantly higher amounts into the skin. However, the 0%, 5%, and 25% PLGA formulations delivered a lower amount of TS into the skin (values in Table 3).

## 4. Discussion

### 4.1. Parameters for the Development of Film-Forming Polymeric Solutions

Limitations of conventional formulations for topical drug delivery include poor adherence to the skin, poor permeability, and a low compliance rate [17]. Bioadhesive film, because it is an intermediate between transdermal patches and semisolid dosage forms, has the advantages of both systems, such as transparency, no stickiness, convenience, less frequent dosing, sustained drug release, and resistance to wiping off. In addition, incorporating cosmetic or therapeutic agents is more convenient with this system than conventional topical drug delivery systems, owing to lower potential loss of the formulation by rubbing.

First, the most important parameters for polymeric solutions are the polymers. Suitable polymers for successful film forming systems may possess transparency, flexibility, and drug encapsulation ability at moderate temperature. To develop a suitable formulation to decrease the frequency of application of topical analgesics, common film-forming polymers, such as hydroxypropyl, methylcellulose, polyvinyl pyrrolidine, and acrylate copolymer, were searched in the literature. However, many of the polymers were hydrophilic and might not provide water resistance in daily life. PLGA was chosen to be incorporated in the polymeric solution because of its many benefits, such as its water resistance, nonirritating and nonallergic qualities, and capability for drug incorporation [15]. To provide an efficient local drug delivery system, Eskandari et al. have investigated the use of butyl-2-cyanoacrylate. To ensure good adhesion of the film to the skin, CA, which is compatible with PLGA, was chosen. CA gets stiffened by polymerization process in the presence of moisture [18,19,20]. When the polymeric film is applied on the skin, the film is formed upon polymerization of PLGA and CA due to the moisture of the skin. Polymerized CA is an excellent polymer candidate for drug delivery because it is a biodegradable, hemostatic, nonallergenic tissue adhesive with local antibacterial properties, and it is inexpensive and widely available [18,19,21]. In addition, it is commonly used and is acceptable to the public, owing to its use in liquid bandage formulations [22]. Currently, CA is used in surgical and clinical adhesives in various surgical procedures such as the treatment of arteriovenous malformation, retinal ruptures, and skin graft placements [23,24]. Eskandari et al. have shown that CA can successfully incorporate drugs for slow release of antibiotics to specific areas without causing an inflammatory response [25].

Solvents play an important role in film formation. The solvent used in the film forming system is responsible for drug-polymer solubility and also affects drug permeation. Some common solvents used in polymeric solutions are ethanol, ethyl acetate, propylene glycols, and acetone [26]. The combination of the PLGA and the adhesive was tested for dissolution in different solvents such as acetone, ethyl acetate, and ethanol. All the ingredients were dissolved readily in acetone and formed a film upon application to the skin. Acetone is approved in use of topical formulation per the inactive ingredient list of FDA [27]. Also, a toxicology report by US. Department of Health and Human Services reported that dermal exposure to acetone did not affect the human health negatively [28].

Apart from the polymer and solvent, other excipients such as plasticizers must be added into the formulation. Plasticizers are low molecular weight resins that may interact with polymer chains and affect polymer-polymer bonding [29]. These interactions may affect film flexibility as well as the permeability toward drug substances. The commonly used plasticizers in polymeric solutions include fatty acid esters, glycol derivatives, phthalate esters, and phosphate esters [30]. The plasticizer used should be compatible with the polymers and should have low skin permeability. Hence, choosing a proper plasticizer is important for the polymeric matrix. In addition, determining the right amount of plasticizer is critical for film-forming polymeric solutions. After the selection of two polymers to form the polymeric solution, proper plasticizers were screened for tolerance to mechanical stress, which may be exerted on the formed film by the movement of the skin. In selecting an appropriate plasticizer, compatibility with the polymers and plasticization efficiency were considered. After application on dermatomed porcine skin, the acetone evaporated, and a thin transparent film was formed in situ. Based on the results, PEG 400 was incorporated as a plasticizer to enhance the mechanical properties and flexibility of the film.

### 4.2. Characterization Methods for Film-Forming Polymeric Solutions

In a transdermal delivery system, drug solubility in the polymer matrix system is a critical factor that may affect the rate and extent of drug permeation [31]. If a drug is not soluble in a polymer matrix, it will become supersaturated and hence unstable. Moreover, when a drug is successfully incorporated in a polymeric matrix, many studies have reported that the stability of the drug improves [32,33]. PLGA formulations are widely used in transdermal and dermal delivery and have a safety profile showing a lack of skin irritation and uniform drug dosing [15,34].

The drug was successfully dissolved in the polymeric matrix, and the absence of crystallization, which might hinder drug permeation into the skin, was verified. In patch development, to improve the permeation and release profiles of drugs, the investigation of the supersaturated state is an important step. A possible strategy to study the solubility of a drug is to disperse it uniformly into a polymeric matrix to prevent drug crystallization [35]. Different techniques have been described to determine the solubility of drugs in polymer matrixes [31,36,37]. Such solubility testing can be time consuming and difficult. Among different techniques, Jain et al. have compared two different methods to predict drug solubility in the polymeric matrix: differential scanning calorimetry and slide crystallization study [31]. In that study, the author found that the experimental solubility potential and the theoretical values from a solubility calculator were similar. In addition, low drug solubility in the drug-polymer matrix was shown to negatively affect drug release. The author concluded that simple slide crystallization was able to predict the saturation solubility of a drug in the polymer. In the present study, after complete evaporation of solvents from our drug-polymeric matrix, crystallization of the drug was not observed under a light microscope, and we confirmed the drug solubility and stability in the system. The complete TS solubility in the polymer matrix system after evaporation of acetone was studied to ascertain that the stability and solubility of TS was not a factor affecting the rate and extent of drug permeation.

The amount of residual solvent was also measured and calculated. Instant solvent evaporation and complete dryness of the formulation was observed after 2 min. As a result, approximately 25% of the residual solvent was left on the application site. Per the International Conference on Harmonisation residual solvent guideline, solvents are classified in the three categories which set limits depending on the toxicity data for each solvent. Acetone is categorized as a Class III solvent, which has a low toxic potential and is safe to use in human [38]. Furthermore, Baino et al. reported that the drug concentration increases upon evaporation of the solvent, which may result in a greater driving force to deliver the drug into the system [39]. However, this variable was eliminated by keeping the solid content of all the formulations constant. Thus, residual solvent was not a factor that led to differences in the permeation profile of TS from the formulations tested.

### 4.3. In Vitro Release Study for Different Types of PLGA

In this study, the release profiles of TS from different PLGA polymers were determined to study the effects of the PLGA parameters on the drug release kinetics. In terms of developing a polymeric system, knowing the release mechanisms and the physicochemical properties is important. The two main release mechanisms related to drug release from a PLGA-based system are degradation and diffusion. Often, the release rate is initially determined by diffusion followed by degradation controlled at the final stage [40]. PLGA systems commonly release the drug in a bi-phasic or a tri-phasic pattern. In this study, for higher molecular weight PLGA polymers, we observed a bi-phasic profile of drug release, with a burst release followed by relatively slow diffusion. Lower molecular weight PLGA groups showed a monophasic release profile, which is preferable for drug delivery systems because it follows a zero-order release profile [41]. The formulation with higher molecular weight compounds 50-8A and 75-5A released almost 40% after 1 h, thus indicating dose dumping, whereas the release profiles for 50-2A and 50-5A showed a gradual release until 72 h. In this study, the film was submerged in the receptor solution, which was more hydrophilic than the polymeric solution. Because the drug was hydrophilic, it may be preferentially located in the receptor solution over the PLGA system. Thus, the higher molecular weight, and hence more lipophilic PLGA, resulted in a more rapid release of the drug than was observed in the groups with lower molecular weight. Based on the result of the release study, 50-2A was chosen for the final formulation to avoid dose dumping effect as well as provide a gradual release of TS until 72 h.

### 4.4. In Vitro Permeation Study

In vitro permeation studies, using the vertical Franz diffusion cell model, have been commonly used to evaluate drug delivery into and across the skin, and is well established as a reliable tool [42,43]. In addition, FDA recommends and endorses the use of an in vitro permeation test to evaluate topical products. FDA guidelines have included the merits of this approach and have established a strong correlation between the in vitro permeation test (IVPT) and in vivo bioavailability data with narrow inter- and intra-variability between data [44,45,46].

#### 4.4.1. Effects of Plasticizers

Plasticizers are important components of film forming systems because they improve the appearance of the film, prevent film cracking, increase film flexibility, and confer desired mechanical properties [47]. By selecting an appropriate plasticizer and optimizing its concentration in the formulation, the release rate of a therapeutic compound can be controlled. To observe the effects of the plasticizer, we kept the formulation ratio between the polymer and the adhesive at 4 to 1 (PLGA:CA), and the same amounts of other components were included, i.e., solvents and drug concentrations. The ratio of plasticizer was increased from 0 to 4 (0%, 5%, 8.75%, and 11.25% *w*/*w*). The polymeric films without any plasticizer and 11.25% *w*/*w* plasticizer delivered a significantly lower amount of TS after 72 h, than did those with 5 and 8.75% *w*/*w* PEG 400. The films with a plasticizer concentration of 8.75% *w*/*w* did not significantly improve the diffusion of the drug in the receptor (as compared with the results with 5% *w*/*w* plasticizer concentration). However, the amount of PEG 400 showed a linear relationship up to 8.75% *w*/*w* with regarding TS delivery into the skin. The formulation with 11.25% *w*/*w* did not show a significant increase over 8.75% *w*/*w* regarding TS delivery into the skin. Hence, 5% *w*/*w* was considered the optimum concentration for the plasticizer. These results may have been due to a polymer-plasticizer interaction that affected the release of the drug from the system. Barhate et al. have studied the carvedilol permeation profile with and without PEG 400 as a plasticizer and have found that the incorporation of PEG 400 increases the film flexibility and permeation rate [48]. The results suggested that too little or too much plasticizer in the polymeric solution can negatively affect the permeation profile of TS. In formulating a polymeric solution, minor variations might be acceptable; however, major changes in the composition should be carefully considered, because they might unfavorably affect the properties of the film forming system and consequently the mechanical or cosmetic performance in drug delivery into the skin.

#### 4.4.2. Effects of Different Types of PLGA

PLGA is one of the most commonly used biodegradable polymers in the field of biomedical devices because of its favorable degradation characteristics and the possibility for sustained drug delivery. It can be engineered to control drug release by changing the polymer molecular weight and ratio of lactide to glycolide. Commercially available PLGA combinations are 50:50, 65:35, 75:25, and 85:15 (LA:GA) in different molecular weights [49]. These physical properties affect the solubility, glass transition temperature, and inherent viscosity, thus resulting in tensile strength and polymer chain flexibility. These parameters help delivery systems or medical devices achieve the desired dosage and release interval. However, evaluation of the system is required to minimize the potential toxicity from dose dumping, inconsistent release, and drug-polymer interactions [50]. In this study, four different types of PLGA were chosen and incorporated in the polymeric solution. Three formulations were 50:50 (LA:GA) with different molecular weights. One of the formulations was 75:25 (LA:GA). There was no significant difference among formulation groups with the same ratio of LA:GA, but the group with a higher proportion of lactide delivered less TS after 72 h. Moreover, the amount of drug delivered into the skin did not show significant differences across all four groups. PGA is a hydrophilic and highly crystalline polymer with a relatively fast degradation rate. Although PGA and PLA are similar structurally, they exhibit different physicochemical properties because of the presence of a methyl group on the alpha carbon. Delayed release of the drug from the higher ratio of LA delivered fewer drugs to the receptor at the end of the study. However, the same ratio with different molecular weights did not show a difference in drug delivery to the receptor and skin. Farahani has investigated the degradation mechanism of PLGA (50:50) and has reported that up to eight weeks are required for complete degradation [51]. In this study, drug diffusion was found to be important factor affecting in vitro release of the drug from the formulation; hence the rate of diffusion influenced drug delivery into and across the skin. Three formulations with 50:50 (LA:GA, different molecular weights) did not result in a difference in permeation profile of TS into and across skin; however, the formulation with 75:25 (LA:GA) delivered a significantly lower amount of TS due to its slower diffusion rate.

#### 4.4.3. Effects of Different Amounts of PLGA

To test the effects of the amounts of PLGA in TS permeation profiles, we varied the amount of PLGA without changing the ratio between CA and plasticizer. The amount of PLGA was increased from 0% to 25% *w*/*w*. Formulations with 20% and 25% PLGA delivered significantly higher amounts than the other groups. Formulations with lower amounts of PLGA required more CA. CA has been used as a synthetic adhesive for tissue adhesive application since the 1980s. CA polymerizes rapidly in the presence of weak basic conditions, such as in water. The interaction between skin and the polymer results in the impressive adhesive strength of CA. Moreover, CA is superior to rival polymers in terms of its strong wet adhesion, drug incorporation, and rapid curing [52,53,54,55]. The trend in permeation profile was observed because the drug’s affinity toward CA was greater than that toward PLGA. Although CA has good adhesion to skin, the rapid polymerization of CA is associated with heat dissipation at the application site, and the brittleness can be problematic [56]. PLGA content was increased while the amount of CA was reduced proportionally to maintain a similar solid content of all formulation. The cumulative drug amount linearly increased with an increase in the amount of PLGA in the formulation. In the optimized formulation, 20% *w*/*w* PLGA was added, resulting in the highest delivery after 72 h without compromising its adhesive property. In this study, an in-situ film forming system was developed by minimizing the CA component and maximizing the amount of PLGA in solution, thus enhancing the delivery of TS into and across the skin.

## 5. Conclusions

Film forming solutions were successfully formulated with polymers from different chemical groups such as PLGA and polymerized CA. These formulations contained a combination of polymers, a volatile solvent, and other optional excipients such as plasticizers, and fixed concentrations were used for all excipients involved. The optimized formulation also enhanced the delivery of TS into and across the skin.

## Figures and Tables

**Table 1:** Properties of the utilized PLGA polymers.

PLGA Polymer	Lactide/Glycolide Molar Ratio	End Group	Molecular Weight (Indicative Range, kDa)
EXPANSORB^®^ DLG 50-2A	1:1	–COOH	5–20
EXPANSORB^®^ DLG 50-5A	1:1	–COOH	42–65
EXPANSORB^®^ DLG 50-8A	1:1	–COOH	80–130
EXPANSORB^®^ DLG 75-5A	3:1	–COOH	37–84

**Table 2:** Composition of the distinct formulations (% (*w*/*w*)).

	Different Types of PLGA				Different Amounts of PEG 400				Different Concentrations of PLGA				
	F1	F2	F3	F4	F1′	F5	F6	F7	F1″	F8	F9	F10	F11
Code	50-2A	50-5A	50-8A	75-5A	4:1:1	4:1:0	4:1:2	4:1:3	20%	0%	5%	10%	25%
Polymer	50-2A	50-5A	50-8A	75-5A	50-2A	50-2A	50-2A	50-2A	50-2A	50-2A	50-2A	50-2A	50-2A
PLGA	20	20	20	20	20	24	17	15	20	0	5	10	25
Cyanoacrylate	5	5	5	5	5	6	4.25	3.75	5	15	12.5	10	2.5
Plasticizer													
PEG 400	5	5	5	5	5	0	8.75	11.25	5	15	12.5	10	2.5
Solvent													
Acetone	69	69	69	69	69	69	69	69	69	69	69	69	69
Drug													
Trolamine Salicylate	1	1	1	1	1	1	1	1	1	1	1	1	1

**Table 3:** Amounts of TS extracted after 72 h in the epidermis and dermis and the total amount of TS for all groups.

	Groups	Epidermis (μg/cm^2^)	Dermis (μg/cm^2^)	Total (μg/cm^2^)
Different types of PLGA	50-2A (F1)	10.4 ± 1.4	1.5 ± 0.4	11.9 ± 1.7
	50-5A (F2)	12.6 ± 2.5	1.3 ± 0.4	13.9 ± 2.4
	50-8A (F3)	8.6 ± 1.3	1.1 ± 0.4	9.8 ± 1.5
	75-5A (F4)	8.7 ± 0.5	1.0 ± 0.2	9.7 ± 0.7
Effect of plasticizer	4:1:1 (F1’)	8.6 ± 0.6	1.6 ± 0.3	10.1 ± 0.6
	4:1:0 (F5)	2.5 ± 0.2	0.3 ± 0.1	2.7 ± 0.3
	4:1:2 (F6)	14.3 ± 2.5	1.9 ± 0.5	16.2 ± 2.8
	4:1:3 (F7)	14.2 ± 0.7	3.7 ± 0.6	17.9 ± 1.0
Different amount of PLGA	20% (F1″)	9.1 ± 2.0	18.2 ± 4.0	27.4 ± 6.0
	0% (F8)	6.3 ± 0.6	0.40 ± 0.1	6.7 ± 0.5
	5% (F9)	9.9 ± 1.0	0.62 ± 0.2	10.5 ± 1.2
	10% (F10)	11.5 ± 1.5	22.9 ± 3.0	34.4 ± 4.5
	25% (F11)	9.5 ± 0.8	1.9 ± 0.4	11.4 ± 1.1
